# Supervised learning reveals circulating biomarker levels diagnostic of hepatocellular carcinoma in a clinically relevant model of non-alcoholic steatohepatitis; An OAD to NASH

**DOI:** 10.1371/journal.pone.0198937

**Published:** 2018-06-26

**Authors:** Anne Hwang, Christopher Shi, Edward Zhu, Farha Naaz, Ping Zhou, Zainab Rasheed, Michelle Liu, Lindsey S. Jung, Bin Duan, Jingsong Li, Kai Jiang, Latha Paka, Satishkumar V. Gadhiya, Dibyendu Dana, Quaisar Ali, Michael A. Yamin, Itzhak D. Goldberg, Prakash Narayan

**Affiliations:** Department of Preclinical Research, Angion Biomedica Corp., Uniondale, New York, United States of America; The University of Texas at El Paso, UNITED STATES

## Abstract

Although cirrhosis is a key risk factor for the development of hepatocellular carcinoma (HCC), mounting evidence indicates that in a subset of patients presenting with non-alcoholic steatohepatitis (NASH) HCC manifests in the absence of cirrhosis. Given the sheer size of the ongoing non-alcoholic fatty liver disease (NAFLD) epidemic and the dismal prognosis associated with late-stage primary liver cancer there is an urgent need for HCC surveillance in the NASH population. Using serum levels of HCC biomarkers as vectors and biopsy-proven HCC or no HCC as outputs / binary classifier, a supervised learning campaign was undertaken to develop a minimally invasive technique for making a diagnosis of HCC in a clinically relevant model of NASH. Adult mice randomized to control diet or a fast food diet (FFD) were followed for up to 14 mo and serum level of a panel of HCC-relevant biomarkers was compared with liver biopsies at 3 and 14 mo. Both NAFLD Activity Score (NAS) and hepatic hydroxyproline content were elevated at 3 and 14 mo on FFD. Picrosirius red staining of liver sections revealed a filigree pattern of fibrillar collagen deposition with no cirrhosis at 14 mo on FFD. Nevertheless, 46% of animals bore one or more tumors on their livers confirmed as HCC in hematoxylin-eosin-stained liver sections. In this training set, receiver operating characteristic (ROC) curves analysis for serum levels of the HCC biomarkers osteopontin (OPN), alpha-fetoprotein (AFP) and Dickkopf-1 (DKK1) returned concordance-statistic/area under ROC curve of ≥ 0.89. Serum levels of OPN (threshold, 218 ng/mL; sensitivity, 82%; specificity, 86%), AFP (136 ng/mL; 91%; 97%) and DKK1 (2.4 ng/mL; 82%; 81%) diagnostic for HCC were confirmed in a test set comprising mice on control diet or FFD and mice subjected to hepatic ischemia-reperfusion injury. These data suggest that levels of circulating OPN, AFP and DKK1 can be used to make a diagnosis of HCC in a clinically relevant model of NASH.

## Introduction

**Fig 1:**
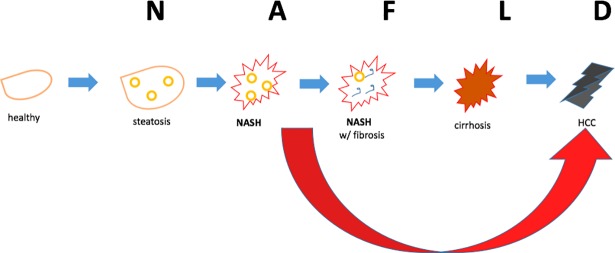
Fatty liver disease continuum. Accumulation of lipid droplets in the liver or steatosis can lead to steatosis+inflammation or NASH which can progress to scarring of the liver or NASH with fibrosis. The next stage cirrhosis is accompanied by significant scarring of the entire liver and presents enhanced risk for HCC. However in the setting of fatty liver disease, HCC can also occur prior to cirrhosis (large red arrow).

Given the diabetes, obesity and metabolic syndrome epidemics, non-alcoholic fatty liver disease (NAFLD) has reached epidemic proportions [1–3]. Left untreated, NAFLD, which starts as simple steatosis, can progress (Fig 1) to non-alcoholic steatohepatitis (NASH), NASH with increasing levels of fibrosis, cirrhosis and hepatocellular carcinoma, (HCC). Mounting evidence [4] suggests that NASH can also progress to HCC in the absence of cirrhosis (Fig 1). In fact, data from a number [4–9] of reports indicates that up to 50% of NASH patients presents with HCC without cirrhosis. Despite these emerging statistics and despite the fact that NASH patients routinely present for liver function tests, this cohort is not typically screened for HCC. Although liver biopsy followed by microscopic evaluation remains the gold standard for diagnosing HCC, ultrasonography with or without serum level of the HCC biomarker alpha-fetoprotein (AFP) is used to make a diagnosis [10, 11]. However, a major challenge associated with surveillance in patients with NASH is that obesity and visceral fat compromise the completeness of an ultrasound examination of the liver and conclusions from imaging data can be confounded by the presence of benign incidentelomas including hamartomas [8]. Use of a single biomarker such as AFP also carries the risk of misdiagnosis. Therapeutic strategy and prognosis with any cancer, especially HCC, is linked to early diagnosis; and accurate diagnosis is as important as early diagnosis.

In the present study, supervised learning was adopted to identify levels of circulating HCC biomarkers in a clinically relevant mammalian model [12, 13] of diet-induced NAFLD-NASH-HCC.

## Methods

### Animal models

The animal protocol was reviewed and approved by the Angion Biomedica Corp. NY animal care and use committee. Mice had access to food and water *ad libitum* throughout the course of the in-life protocol.

The training set (n = 76) comprised adult male C57BL/6 mice (20–22 g) randomized to standard lab chow (control; n = 20) or a fast food diet (FFD; n = 56; rodent diet with 40 kcal% fat, 20 kcal% fructose and 2% cholesterol; D09100301, Research Diets, NJ) for up to 14 mo. Animals were sacrificed at 3 mo (n = 8, control; n = 32 FFD) or 14 mo (n = 12 control; n = 24 FFD) into their diets. Each individually tracked animal acted as its own serum and liver control. The test set (n = 32) comprised adult male C57BL/6 mice (20–22 g) randomized to FFD (n = 24) for up to 7 months, adult male Kcnn4/ KCa3.1 null mice (n = 2) [14] on control diet and adult male CD-1 mice (~40 g) on control diet subjected to 30 min normothermic global hepatic ischemia via occlusion of the portal triad [15] under ketamine/xylazine (25/5 mg/kg, intraperitoneal) anesthesia and up to 24 hr reperfusion (n = 4) and adult male CD-1 mice on control diet (control, n = 2).

### Liver histopathology

At sacrifice, livers were examined (gross) for the absence or presence of tumors by two independent observers. Several biopsies were obtained from each liver and fixed in formalin (10%); whenever tumors were present, both tumors and remote liver were biopsied. Hematoxylin-eosin (H&E)-stained liver sections were studied under a microscope by blinded observers for evaluation of both, NAFLD Activity Score (NAS; worsening 0–8 scale; 0–3 steatosis; 0–3 inflammation; 0–2 hepatocyte ballooning) and HCC [16–18]. NAS was averaged from three independent observers whereas HCC diagnosis required confirmation from both gross liver and microscopic observation—broad trabecular growth pattern of atypical hepatocytes and clusters of multinucleated hepatocytes—by 2 trained and independent observers [17, 18]. At sacrifice, total liver hydroxyproline, a surrogate for collagen, was quantified from liver biopsies using a colorimetric assay as described previously [19]. Picrosirius red staining of liver sections followed by semiquantitation (Bioquant) of extracellular fibrillar collagen [19] was performed by a blinded observer. In the hepatic ischemia-reperfusion study, in addition to making a diagnosis for presence or absence of HCC, H&E-stained liver sections were evaluated under a microscope for presence of necroinflammatory damage by blinded observers.

### Serum biomarkers

Commercially available enzyme-linked immunosorbent assay (ELISA) kits were used for determination of serum biomarker levels. In many instances, following a pilot study, serum samples had to be diluted (10–50 fold in saline) and the ELISAs rerun so that sample levels remained within the standard curve. The HCC biomarkers evaluated (catalog number, source of ELISA kit) are as follows: Alpha fetoprotein (AFP) (MAFP00, R&D), Lens culinaris A-reactive fraction of AFP (AFP-L3) (MBS724605, MYBioSource), Des γ carboxyprothrombin (DCP / PIVKA-II, MBS2516006; MYBioSource), Glypican-3 (MBS705612, MYBioSource), Osteopontin (OPN, MOST00; R&D), Golgi protein-73 (GP73, MBS024709; MYBioSource), Dickkopf-related protein 1 (DKK1, DY1765; R&D) Serum cystatin C was measured using an ELISA kit (MSCTC0, R&D). Serum aspartate transaminase (AST) and serum alanine transaminase (ALT) levels were measured by a commercial laboratory (Northwell Health, NY).

### Data analysis

Data are presented as mean ± standard error or mean. Student’s T-test or one-way analysis of variance followed by Tukey’s post-hoc test was used to compare data between groups. A p<0.05 was deemed significant. Receiver operating characteristic (ROC) curves were generated in Excel using Pivot Tables and area under ROC (AUROC) calculated using the trapezoidal method. For AUROCs ≥0.8 (good-excellent range), sensitivity (S_n_), specificity (S_p_) and cutoffs/thresholds were calculated.

## Results

### Training set

#### NAFLD-NASH-Liver tumors

**Fig 2:**
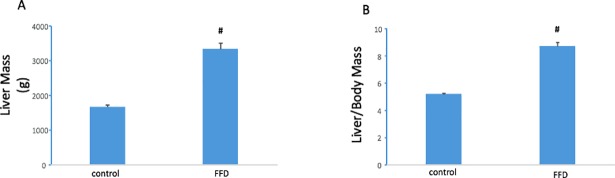
Hepatic phenotype on FFD. Compared to livers from animals on a control diet for 3 mo, livers from FFD (3 mo) animals had larger mass and liver-to-body mass ratio (#, p < 0.01 vs. control).

**Fig 3:**
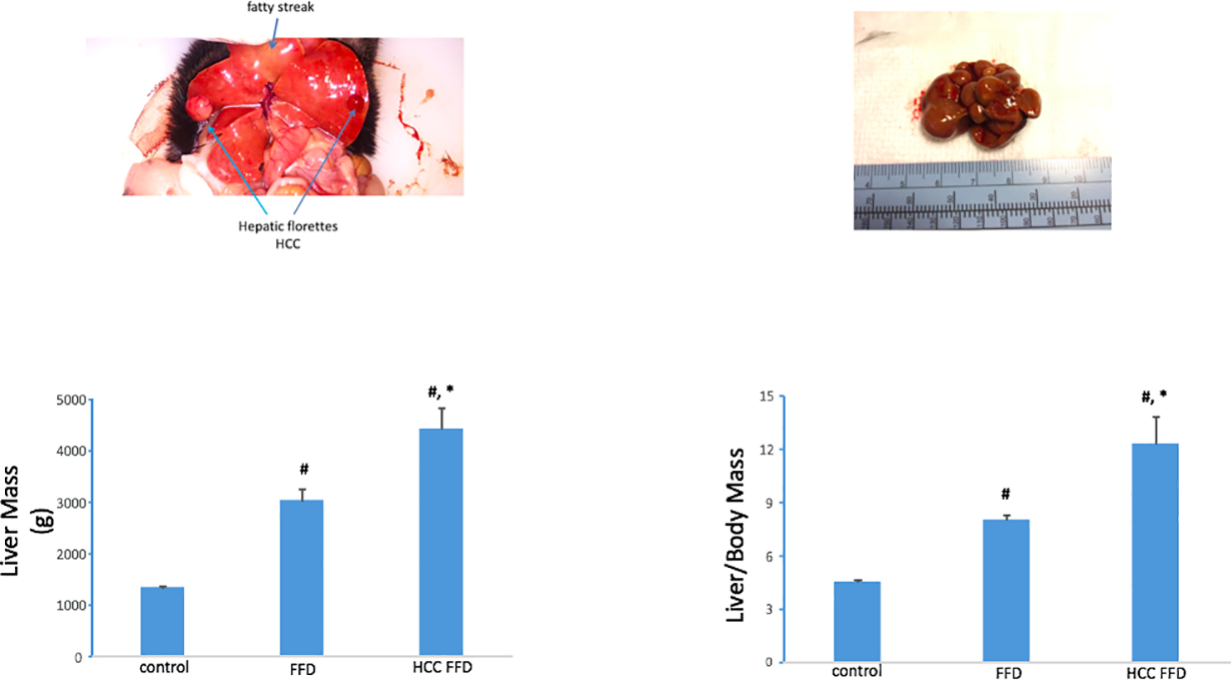
FFD-induced HCC. Of the 76 animals entered into the training set, 65 animals bore no tumors on the liver. Of the 24 animals on FFD for 14 mo, 11 exhibited liver tumors which manifested as one or more polyp-like structures on the liver. Animals on FFD for 14 mo had larger liver and liver to body mass ratios compared to the control diet cohort. Animals with liver tumors (labeled HCC FFD) had the highest values for these endpoints (#, p < 0.01 vs. control; *, p < 0.01 vs. FFD).

**Fig 4:**
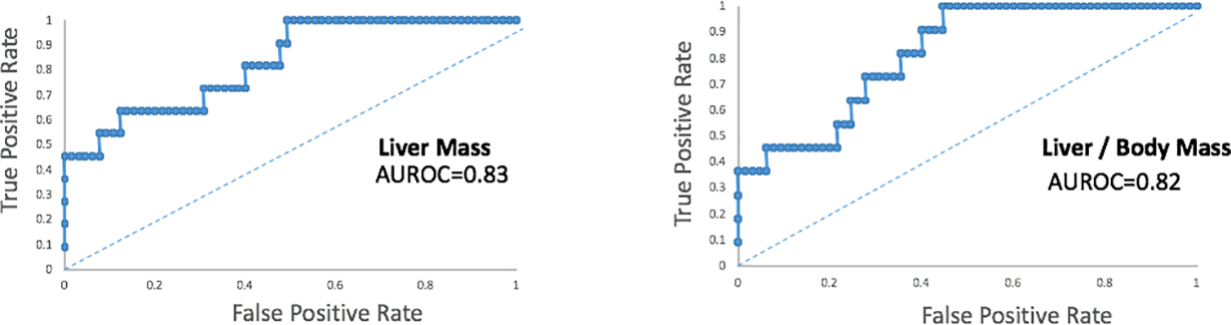
ROC curves for liver mass and liver-to-body mass. ROC curves showing the performance of liver mass as a diagnostic for HCC (AUROC 0.83) and liver-to-body mass ratio for HCC (AUROC 0.83). Data from the entire training set of 76 animals were used for plotting these curves.

In the training set,the FFD cohort exhibited a distinct fatty liver phenotype by 3 mo. Livers were relatively pale and spongiform in appearance and liver mass and liver-to-body mass ratios were increased compared to the 3 mo control diet cohort (Fig 2). Determination of liver function tests at 3 mo demonstrated elevated AST (142±6 vs.69±17; p<0.01) and elevated ALT (110±15 vs.47±9; p<0.05) in the FFD vs. control diet cohort. Of the 24 animals that had been randomized to FFD for 14 mo, 11 animals, i.e. 46% exhibited liver tumors (Table 1 and Fig 3). Furthermore, these liver tumor-bearing animals had the highest liver mass and liver-to-body mass ratio (Fig 3). Grouping subjects as HCC-free or HCC. i.e. binary outcome, ROC curve analysis for liver mass and liver-to body-mass ratio yielded AUROCs of 0.83 and 0.82, respectively (Fig 4).

**Table 1:** Grouping of training set animals based on type of diet, duration on diet and absence or presence of liver tumors.

n	Diet	Duration on Diet	Tumor(s)
8	Control	3 mo	No
32	FFD	3 mo	No
12	Control	14 mo	No
13	FFD	14 mo	No
11	FFD	14 mo	Yes

#### Liver histopathology

**Fig 5:**
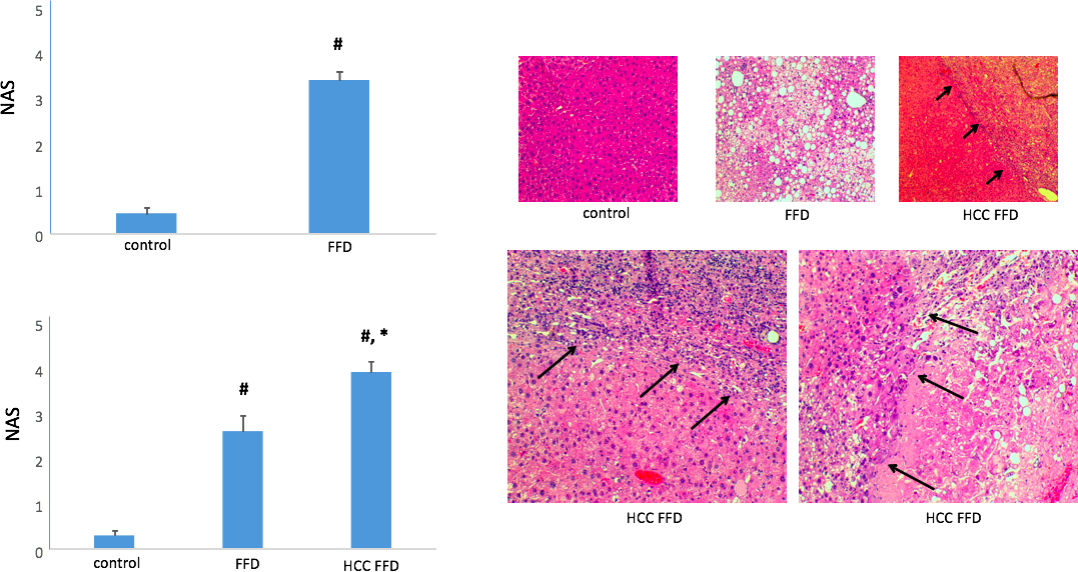
Liver histopathology. (Top left) At 3 mo on the diets, NAS was elevated in the FFD cohort vs. the control diet cohort (#, p < 0.01 vs. control). (Bottom left) At 14 mo on the diets, NAS was elevated in the FFD cohort vs. the control diet cohort (#, p < 0.01 vs. control). Animals exhibiting liver tumors (HCC FFD) exhibited highest NAS (*, p < 0.01 vs. FFD). (Top right) Representative H&E stained liver sections (10 X) from the 14 mo control, FFD and HCC FFD cohorts are shown. Steatosis was prominent in the FFD cohort. tTe HCC FFD cohort showed a trabecular growth pattern of atypical hepatocytes and clusters of multinucleated hepatocytes with a distinct margin (arrows) between the cancerous and non-cancerous parenchyma. (Bottom right) Representative H&E stained liver sections (20 X) from HCC FFD livers showing aspects of NASH (steatosis and inflammation) and HCC (arrows).

To characterize disease at the microscopic level, H&E stained liver sections from the different time and diet groups were examined. The FFD cohort showed hallmark characteristics of NASH evidenced by increased NAS (Fig 5) at both 3 mo and 14 mo. Within the 14 mo FFD cohort, livers that presented with tumors on gross observation presented with a trabecular growth pattern of atypical hepatocytes and clusters of multinucleated hepatocytes. In fact, a distinct margin (see arrows) was observed between the cancerous and non-cancerous parenchyma. Within the 14 mo cohorts, livers characterized as HCC positive had the highest NAS (Fig 5).

**Fig 6:**
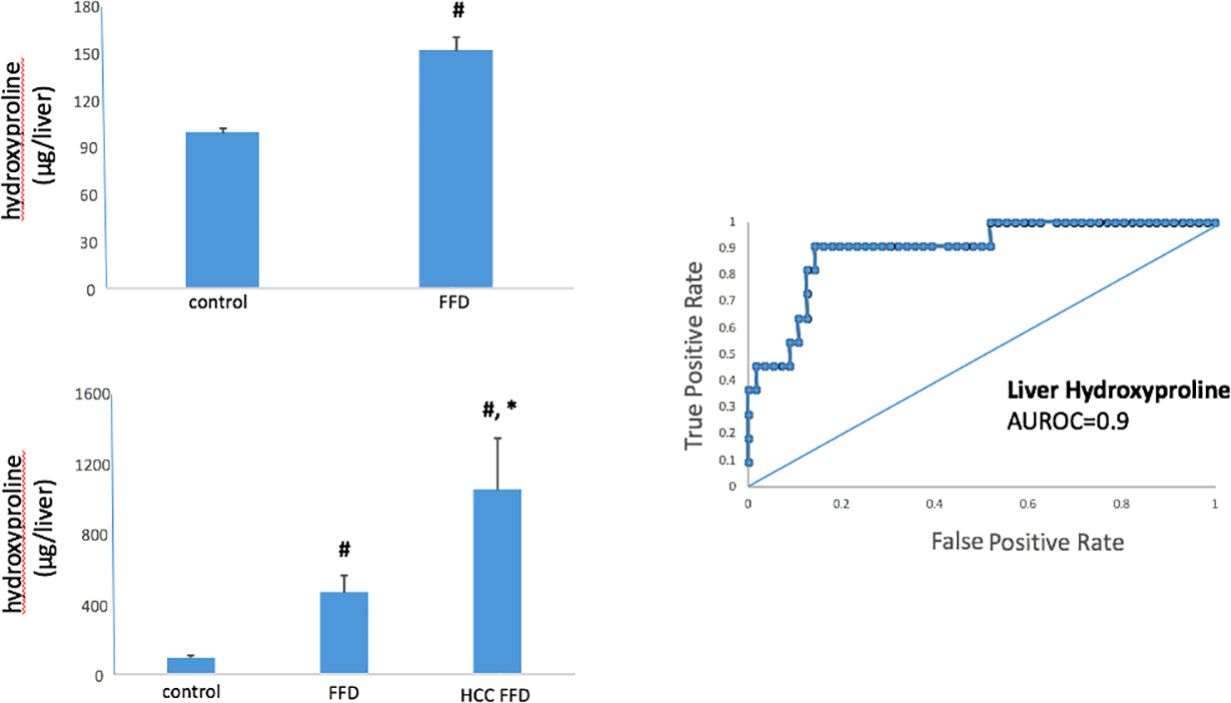
Liver hydroxyproline. (Top left) Animals randomized to FFD for 3 mo exhibited ~ 50% higher liver hydroxyproline content compared with animals randomized to a control diet for an equivalent time period (#, p < 0.01 vs. control). (Bottom Left) Animals on FFD for 14 mo but free of HCC had >4-fold higher liver hydroxyproline content than animals on a control diet for the same time interval (#, p < 0.01 vs. control). Liver hydroxyproline content was highest in the 14 mo HCC FFD cohort (*, p < 0.01 vs. FFD). (Right) ROC curve for liver hydroxyproline as a diagnostic for HCC yielded an AUROC of 0.9.

Next, we determined whether this model of NASH is accompanied by scarring or liver fibrosis. Animals randomized to FFD for 3 mo exhibited 50% higher liver hydroxyproline content, a marker of fibrosis, compared with animals randomized to the control diet (Fig 6). Animals on FFD for 14 mo but free of HCC had >4-fold higher liver hydroxyproline content than animals on a control diet for the same time interval (Fig 6). Liver hydroxyproline content was highest in the 14 mo HCC FFD cohort, 10-fold greater than the 14 mo control diet cohort. Grouping subjects as HCC-free or HCC, ROC curve analysis for liver hydroxyproline yielded an AUROC of 0.9 (Fig 6).

**Fig 7:**
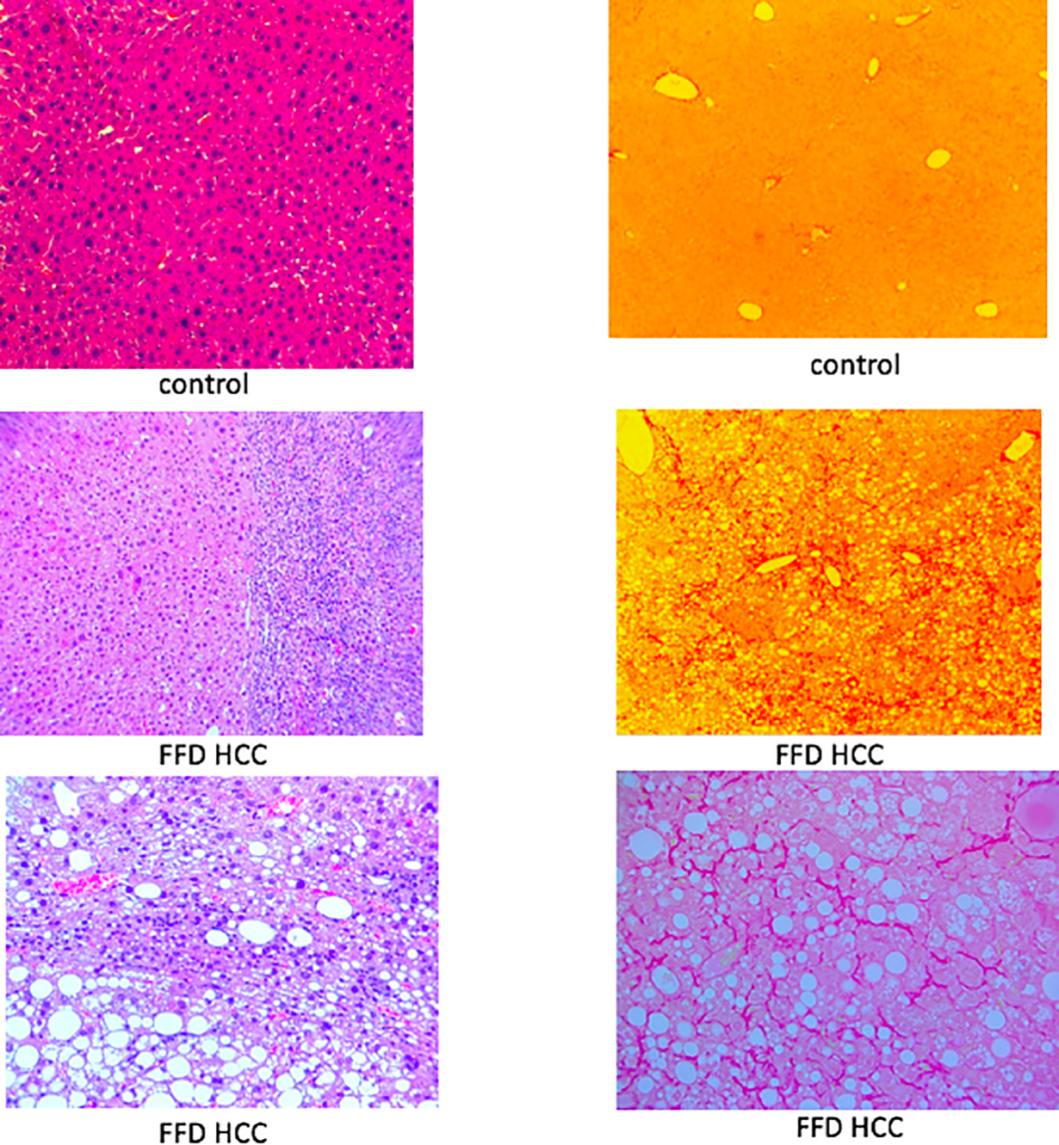
FFD model of NASH with fibrosis. (Top) Representative H&E (left) and Picrosirius red (right) stained liver sections (10 X) from an animal on a control diet for 14 mo. (Middle) Representative H&E (left) and Picrosirius red (right) stained liver sections (10 X) from an HCC FFD animal that was on FFD for 14 mo. (Bottom) 20 X section from that animal (left, H&E; right Picrosirius red). While a filigree pattern of Picrosirius red staining was observed in the HCC FFD cohort, there was no histological evidence of cirrhosis.

To determine whether the HCC FFD cohort had progressed beyond fibrosis to cirrhosis, a disease state characterized by fibroconnective tissue hypertrophy and pseudolobule formation [20], Picrosirius red-stained sections of livers were examined. While livers from the 14 mo control diet cohort exhibited little, or no Picrosirius red staining, livers from the FFD cohort classified as HCC exhibited a filigree pattern of Picrosirius red staining (Fig 7), with a collagen proportionate area (3±0.7%) consistent with fibrosis but not cirrhosis (typically 25%) [21].

#### HCC biomarkers

**Fig 8:**
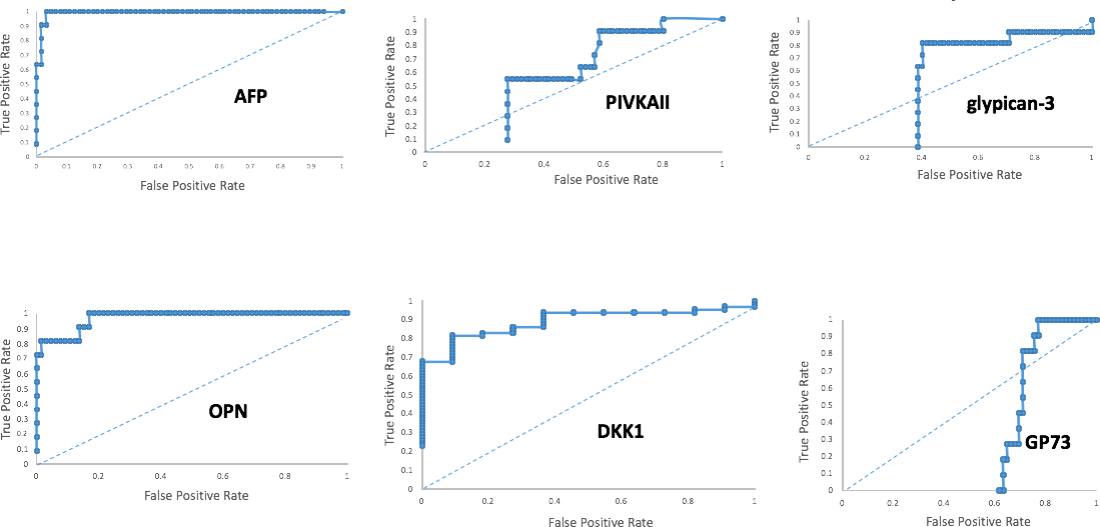
ROC curves for HCC biomarkers. Performance of the biomarkers listed in Table 1 as diagnostics for HCC are shown as ROC curves. Only OPN, AFP and DKK1 had AUROCs ≥ 0.8.

Next, serum levels of cystatin C and the HCC biomarkers listed in Table 1 were measured in mice randomized to control diets or FFD. For ROC analysis, levels of biomarkers were grouped according to the absence of presence of HCC, i.e. binary output, regardless of diet type or time on diet. There was no difference in serum levels of cystatin C between the HCC-free (600±61 ng/mL) and HCC (633±26 ng/mL) animals. On the other hand, there was a significant (p<0.01) difference in serum levels of AFP between the HCC-free (36±4 ng/mL) and HCC (249±23 ng/mL) animals. ROC curves for the HCC biomarkers listed in Table 1 were computed (Fig 8). Serum AFP-L3 levels were consistently below the standard curve and this biomarker was rejected. OPN, AFP and DKK1 had AUROCs > 0.8 for HCC and S_n_, S_p_ and thresholds for these biomarkers were computed (Table 2).

**Table 2:** Diagnostic performance of HCC biomarkers. OPN, AFP and DKK1 each had AUROCs > 0.8 for HCC and exhibited excellent S_n_ and S_p_. Thresholds or cutoffs for each of these biomarkers is listed.

Biomarker	AUROC	Sn (%)	Sp (%)	Threshold (ng/mL)
OPN	0.97	82	86	218
AFP	0.99	91	97	136
DKK1	0.89	82	81	2.4

### Test set

#### Liver histopathology & HCC biomarkers

**Fig 9:**
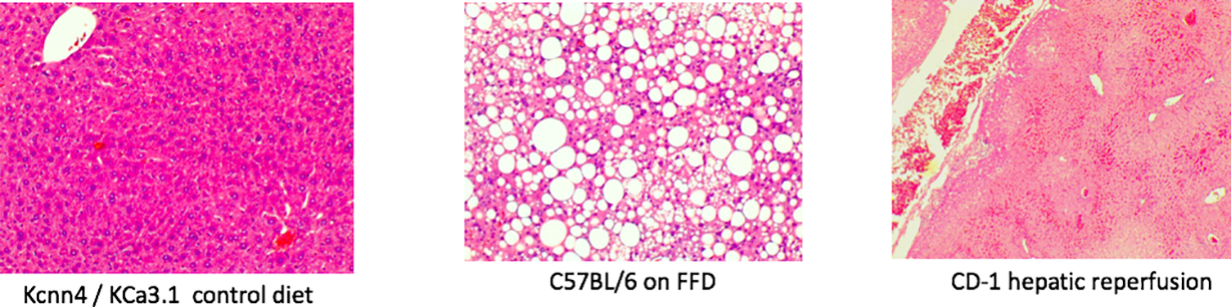
Diagnostic performance of HCC biomarkers in test set. (Top) Representative H&E-stained livers sections (20 X) from a Kcnn4/ KCa3.1 null mice mouse on a control diet (left), a C57BL/6 mouse on FFD for 7 mo (middle) exhibiting steatosis and CD-1 mouse submitted to hepatic ischemia and 24 hr reperfusion (right) exhibiting necroinflammatory injury. There was no evidence of HCC in the test set. (Bottom) Diagnostic accuracy of OPN, AFP and DKK1 in the test set.

Gross examination of livers from the test cohort comprising C57BL/6 mice on FFD for 7 mo or KCa3.1 null mice, CD-1 mice and CD-1 mice subjected to hepatic ischemia-reperfusion injury, all on control diet, revealed no tumors. Microscopic examination of H&E-stained livers also indicated that they were HCC-free albeit their histopathology was consistent with being on a control or fatty diet or subjected to ischemia-reperfusion (Fig 9). Indeed in animals subjected to hepatic ischemia-reperfusion increased in AST (320±67 IU vs. 47±9 IU control; p<0.05) and ALT (283±80 IU vs. 26±4 IU control; p<0.05) were observed.

In parallel, serum levels of OPN, AFP and DKK1 were measured in animals that comprised the test set. C57BL/6 mice submitted to FFD for 7 mo or the KCa3.1 null mice, CD-1 mice or CD-1 mice subjected to hepatic ischemia-reperfusion injury exhibited an average serum level of AFP of 65±5 ng/mL (range 54 to 113 ng/mL),below the HCC threshold/cutoff of 136 ng/mL. Use of AFP as a diagnostic in this test set was associated with 100% accuracy. Measurement of serum OPN in these animals returned an average of 121 ng/mL (range 96–184 ng/mL), below the HCC threshold/cutoff of 136 ng/mL. Use of OPN as a diagnostic in this test set was associated with 100% accuracy. Measurement of serum DKK1 levels in these animals returned values in 28 out of the 32 animals that were consistent with an HCC-free diagnosis. Four of the 32 animals exhibited serum DKK1 levels past the cutoff for HCC. Since none of the animals on the training set had HCC, these 4 subjects tested false positive for HCC. Use of DKK1 as a diagnostic in this test set was associated with 88% accuracy.

## Discussion

In a murine model of FFD-induced NAFLD, animals developed NASH with increasing levels of fibrosis but not cirrhosis. By 14 mo on FFD, a large subset of animals exhibited HCC. Use of a supervised learning approach indicates that the serum HCC biomarkers OPN, AFP and DKK1 are excellent diagnostics for HCC in NASH with AUROCs of 0.97, 0.99 and 0.89, respectively, and thresholds of 136 ng/mL, 218 ng/mL and 2.4 ng/mL, respectively.

HCC is the fifth most common tumor worldwide and the second most common cause of cancer-related death [10, 11]. Historically its incidence has stemmed from the HCV epidemic in the United States, and the hepatitis B virus epidemic in the Far East [10, 11, 22]. Within the continuum of liver disease, cirrhosis carries increased risk for HCC. In fact, the American Association of Liver Disease (AASLD) recommends screening of cirrhotics every 6 months because early detection improves overall survival [10, 11]. It is fully recognized that therapeutic strategy and prognosis in HCC is linked to disease stage, with Barcelona Clinic Liver Cancer (BCLC) stage 0-A patients having better outcomes [23]. The highly vascularized liver makes resection of later stage larger tumors impractical. In the setting of liver cancer metastasis, more often than not, death is the outcome. BCLC Stages B-D are deemed non-curative [23].

Increasing clinical evidence suggests that fatty liver disease also leads to HCC. With ~ 30% or adults in the United States having some steatosis, and 3–12% of adults in the United States presenting with NASH, it is estimated that fatty liver disease-related HCC will overtake all other causes of primary liver cancer with the next 2 or 3 decades [24]. Very worrisome are reports [4] of HCC in patients with NASH but no cirrhosis. In a review [5] of the tumor board database at Brooke Army Medical Center (BAMC) 13% of the HCC cases occurred in non-cirrhotic NAFLD/NASH. In a study [25] by Dyson and colleagues characterizing the demographics of HCC from 2000 to 2010 in the region surrounding Newcastle-upon-Tyne in North East England, ~30% of the HCCs diagnosed occurred in the absence of cirrhosis. In a clinical study [7] by Ertle and colleagues, ~ 42% of individuals with NAFLD-NASH-HCC had no evidence of cirrhosis. Following a comprehensive survey of the clinical literature, Kolly and Dufour reported [8] a 50% prevalence of HCC in the context of NASH without cirrhosis. Perhaps most alarming are findings by Paradis et al [6] of HCC in NASH patients with no fibrosis, and a warning from Cholankeril and colleagues [9] of HCC in pre-NASH NAFLD patients. Given that ~80 million Americans presents with NAFLD, ~16 million people in the United States alone have NASH and ~5 million of those have NASH with advanced fibrosis [24], even a small percent incidence of HCC translates to staggering numbers. It is becoming evident that with aggressive use of antivirals for HCV the risk of progression to cirrhosis and development of HCC secondary to HCV is declining. By contrast, the incidence of NASH-HCC is rising rapidly [9]. Furthermore, patients with NASH-HCC often present with large tumors and have a worse prognosis than those with HCC from other etiologies [8]. A retrospective analysis [26] of the Surveillance, Epidemiology and End Results (SEER)-Medicare database, 50% of viral-hepatitis-infected patients with HCC died after one year, whereas 61% of the fatty liver patients with HCC died after one year. All things considered, there is an emergent need for HCC surveillance in the NASH patient. From the patient’s and loved ones perspective, correct diagnosis of HCC is as important as early diagnosis.

In the present study, we tracked animals randomized to a control or FFD for up to 14 mo in an attempt to simulate, at least, in part, fatty liver disease. The FFD included fat, cholesterol and fructose, a recipe that is associated with steatohepatitis [12, 13]. Indeed, within 3 mo on this diet, animals had developed hallmark characteristics of NASH. Some liver fibrosis, evidenced by a 50% increase in liver hydroxyproline content, was evident at 3 mo on this diet whereas by 14 mo on this diet animals presented with more advanced liver fibrosis. Picrosirius red is a marker of fibrillar collagen and use of this stain demonstrated a filigree network of collagen deposition, consistent with the development of liver fibrosis in this model. A salient and clinically relevant feature of this model is progression to HCC. While HCC has been reported in other models of diet-induced liver disease [27, 28], those models typically combine a chemical such as streptocozocin or diethyl nitrosamine as a means to accelerate and aggravate liver pathology, often resulting in ~100% incidence of HCC in 12–20 weeks. The FFD model described herein is designed to mimic a Western diet and has a more natural progression to HCC, similar to that observed clinically. The other salient and clinically relevant aspect of this model is the appearance of HCC in the absence of cirrhosis. In fact, at 14 mo on FFD, while animals did develop liver fibrosis none had progressed to a cirrhotic phenotype. Absence of cirrhosis was evidenced by the filligree pattern of Picrosirius red staining and is a finding consistent with other reports [12, 13] using fatty diet models. In the present study, of the animals on FFD for 14 mo, 46% developed HCC, a percentage in agreement with some of the clinical reports referenced above. ROC curve analysis showed that HCC is present in animals with increased liver mass, increased liver to body mass ratio and increased liver fibrosis, in as much as liver hydroxproline content is a marker of the same. Clinically, these measurements are of little or no diagnostic value and their thresholds for liver tumors were not computed. Nevertheless, these data suggest that in this murine FFD model NASH progresses to HCC and supports the notion that this model mimics, at least, in part, clinical findings of NASH-related HCC in the absence of cirrhosis.

Liver biopsy-microscopic evaluation remains the gold standard for diagnosis of HCC. Given that seeding of the tumor in the needle tract occurs only in 1–3% of biopsies [29] and taking into account the logistics including need for an interventional radiologist, risks and patient compliance issues associated with liver biopsies, ultrasonography with or without serum alpha-fetoprotein (AFP), an HCC biomarker, is being used to diagnose HCC. Utilization of serum biomarkers is not only playing a major role in surveillance strategies in higher risk for HCC populations such as cirrhotics but also in risk stratification and prediction of recurrence following initial therapy [30–34]. Several serum markers have been identified in addition to AFP for diagnosis of HCC. These biomarkers include a fucosylated isoform of AFP reactive to *Lens culinaris* agglutinin, known as AFP-L3, glypican-3, OPN, GP73, PIVKA-II also known as DCP and DKK1, among others [31–34]. Many of these biomarkers are in clinical testing but other than AFP none has clinical approval. Furthermore, each of these biomarkers, including AFP, suffers from suboptimal S_n_ and S_p_ making its use as a standalone diagnostic unreliable [35, 36]. HCCs are not homogenous–there is widespread intratumoral heterogeneity at both the genomic and epigenomic levels in HCC [37]. Furthermore, there are multiple histologic HCC subtypes [38, 39]. Thus, some HCCs may have normal or only mildly elevated levels of AFP, but high levels of AFP-L3. Similarly, other liver tumors may have high levels of PIVKAII/DCP but normal levels of AFP. Furthermore, levels of many of these biomarkers including AFP are elevated in the setting of steatosis and liver regeneration which can lead to a false positive diagnosis for HCC or not elevated in the setting of smaller tumors leading to a false negative diagnosis. With current data suggesting that no single biomarker alone is likely to have optimal S_n_ and S_p_ for detection of HCC, particularly during early disease, combining biomarkers might represent a mechanism to improve surveillance. In fact, gastroenterologists in Japan and the United Kingdom rely on BALAD-2 and GALAD calculators [33] for diagnosing HCC secondary to hepatitis B and C viruses and alcoholism albeit the scores are a function of disease etiology and are influence by regional differences.

Following a comprehensive survey of the literature, we queried a panel of serum-based HCC biomarkers (Table 1) to develop a diagnostic framework for HCC in a clinically relevant model of NASH. Cystatin C, whose levels have not been reported to change with HCC was used as a control [40]. A training set comprising 76 animals on 2 different diets and 2 different timepoints was used in this study. HCC was diagnosed by the presence of tumors on the liver coupled with histopathological confirmation of disease. Based on ROC analysis, 3 biomarkers, OPN, AFP and DKK1, and their thresholds, were identified with excellent diagnostic potential for HCC in NASH. The other biomarkers, viz. AFP-L3, PIVKAII/DCP, glypican-3 and GP73 performed poorly as diagnostics in this diet-induced model of NASH-related HCC; including the GALAD subset, AFP-L3 and PIVKAII/DCP. Analyzing the reason(s) for their poor performance is beyond the scope of this study albeit clinical use of those biomarkers has typically occurred in the non-NASH setting [33]. As anticipated, cystatin C levels were unaffected by the absence or presence of HCC [40]. Importantly, in a test set comprising different strains of mice, mice on FFD or control diets and mice subjected to hepatic ischemia-reperfusion, use of OPN and AFP were associated with 100% diagnostic accuracy whereas use of DKK1 was associated with 88% accuracy. Our empirical findings led us to a quantitative framework for making a diagnosis of HCC in a model of NASH without cirrhosis, which, to the best of our knowledge, is the first such description.

These findings may be clinically relevant. If each of these 3 tests is positive for HCC in a serum sample, the likelihood of disease is high and can form the basis for triggering a confirmatory test such as imaging. On the other hand, risk for misdiagnosis of HCC (false positive) is distributed amongst 3 potentially independent diagnostics and can therefore reduce the need for additional tests Statistical rules for combining sensitivities and combining specificities are readily available in case of conflicting data from the diagnostic assays following either parallel or serial testing of blood samples [41]. Serum samples can be obtained repeatedly with minimal discomfort to the subject—especially so in a patient population that routinely needs to present for liver function tests every 3–4 months. In addition to their use as a diagnostic these biomarkers can be used to determine the efficacy of treatment strategies and disease resolution. This study sets the foundation for developing a point-of-care HCC diagnostic assay for the NASH patient.

Nevertheless, there are several limitations to these findings. It is possible that very small and/or embedded tumors might have been missed under gross and microscopic observation. Second, it remains to be determined whether these findings are applicable to other models of NASH and to species other than the mouse. Third, tumor burden has not been correlated with biomarker levels. It would be important to conduct a longitudinal study coupling serum biomarker analysis with high resolution liver imaging to determine how early HCC can be diagnosed in the setting of NASH and without the need to sacrifice the animal. It remains to be established whether serum levels of biomarkers recede with decreasing tumor size or with tumor resolution. Finally, the biomarkers queried is merely a subset of the HCC-related biomarkers described in the literature; harnessing additional biomarkers can potentially improve diagnostic performance.

## Conclusion

We have demonstrated for the first time a quantitative relationship between the levels of circulating biomarkers and HCC in a clinically relevant model of NASH without cirrhosis. Further development and clinical validation of this technology could potentially be used to facilitate early and accurate diagnosis of HCC in a burgeoning NASH population.
